# Aphasia and Testamentary Capacity

**Published:** 1898-09-24

**Authors:** 


### APHASIA AND TESTAMENTARY CAPACITY.

The occurrence of aphasia, meaning thereby not merely loss of speech but loss in varying degree of the power of understanding spoken or written language, has raised many questions as to the capacity of persons so afflicted to make a will, and although it cannot be said that the discussion at Edinburgh threw any new light upon this subject, yet the very interesting address by Sir William T. Gairdner served to place what we do know about it in a clear and common sense light, and to wipe away many of the metaphysical cobwebs by which it has been surrounded by some writers. So far as concerns the anatomy of the parts involved, we have to recognise that there are at least four centres of localisation in the brain, lesions of which may affect the power of speech and of written language. There is Broca's convolution, and there is the auditory centre, which play one upon the other, the auditory centre transmitting to Broca's convolution the impressions of spoken words through the ear, while the mechanism of speech are set in motion from Broca's speech centre in the brain; so that these two centres are concerned chiefly with the reception and the production of vocal speech.

Then there are two centres which are connected with graphic language, with written or printed words, centres again which, so far as the mind is concerned, probably work through the auditory centre. There is, no doubt, a fifth centre having to do with the ideation of the word, as apart from the mechanism of its production, but this is not definitely localised. It is, therefore, sufficient to take into account the ingoing and the outgoing mechanisms, and to divide the former into those that enter by the ear and by the eye, and the latter into those that express thought by speech and by writing.

The matter is, of course, constantly complicated by the fact that the lesion by which any one or more of these centres may be injured is almost certain at the same time to have done other damage to the brain, the extent of which is in no way indicated by the amount of interference with speech which may exist in any given case.

A question, however, which more or less overhangs the whole problem is the general one as to the mental state of aphasics—how far, that is, a man who has lost his words retains his reason. That words are essential to the development of all higher mental development may well be admitted; nay, it may be conceded that a man who had never been otherwise than aphasic, who has, that is, been congenitally deficient in any of the mechanisms which go to make the speech faculty, could never become a reasoning animal and could not rise much above the level of a dog, an elephant, or a horse.

But when we come to the case of a man who is by a sudden accident lamed as regards the mechanism of this particular faculty, having all his reasoning processes well-developed beforehand, we cannot admit that he of necessity suffers any derogation at all of the higher faculties merely because he has lost that of speech; although practically he may suffer in almost any degree in consequence of other faculties besides that of speech being involved in the accident in question—as, for example, in the extreme instance in which an aphasic is also rendered comatose.

If, then, we come back to the question how far does this laming of one faculty interfere with a man's power of making a will, we see that it is a question which does not admit of an answer in general terms, and in fact requires a consideration of the further question what amount of intellect it requires to make a will, even in cases that are not aphasic.

In regard to this it has to be borne in mind that, although a will may be a very complex document, it may on the other hand be very simple and very easy to comprehend, so that when one is asked whether an individual had mind enough to make a will we immediately retort, What will? For while even a moderately smart man might be puzzled by the phraseology of some forms of will, a comparatively low intelligence might quite comprehend so simple a formulary as that adopted by the late M. Pasteur, "I give all my goods to my wife."

Granting, then, that the existence of aphasia imposes on a man a difficulty in giving expression to his thoughts, and that the malady in connection with which aphasia occurs may or may not interfere with his capacity for understanding the contents of the will, we have, in deciding the capacity of any given aphasic person to make a will, to bear in mind (1) the nature of the will which it is sought to establish, (2) the evidence which may be offered of the persistence of mental capacity to understand such a document, and finally the evidence which exists as to his power of communicating his assent to or his dissent from the proposals it may contain.

A completely aphasic, but not otherwise insane or stupid person, has no doubt, as regards his inner mind, the full capacity of making a will of some kind, but the question whether an individual aphasic was capable of making the individual will which he is asserted to have made, is one which must be submitted to a jury and be fought out in a court of law, not upon abstract principles at all, but on the basis of the individual facts of the particular case.

